# Peacock’s Bromides

**Published:** 1892-02

**Authors:** 


PEACOCK'S BROMIDES.—Dr. R Robbins, of Hartford, Kan., writes that "this is a most excellent preparation; has used it with success in spasms, nervousness, etc.; that it is an excellent remedy for headaches; and adds that he cannot get along without it."

